# Cognitive Function in Sexual and Gender Minority Older Adults

**DOI:** 10.1093/geroni/igaa057.2442

**Published:** 2020-12-16

**Authors:** Jason Flatt, Samantha John, Paula Frew

**Affiliations:** 1 University of Nevada, Las Vegas, School of Public Health, Las Vegas, California, United States; 2 University of Nevada, Las Vegas, Las Vegas, California, United States; 3 University of Nevada, Las Vegas, School of Public Health, Las Vegas, Nevada, United States

## Abstract

Nearly 3.5 million sexual and gender minority (SGM) adults aged 60+ in the U.S. identify as lesbian, gay, bisexual, transgender, and/or queer. We recruited over 50 diverse SGM older adults from the community to better understand correlates of their cognitive function. The Telephone Interview for Cognitive Status, an 11‐item screening test of global cognition was used over the phone or in-person. We will describe relationships among cognition and several sociodemographic and health variables (age, sex assigned at birth, SGM identities, race/ethnicity, and health). Past research has highlighted higher rates of perceived memory problems among lesbian, bisexual and transgender adults compared to both gay men and heterosexual men and women. These rates were also higher among those who identify as women. We highlight implications for researching gender identity and cognition in late life, such as the influence of gender roles on cognition and the assessment of gender expression and related constructs.

